# Supplementary material for: Peripheral helper-T-cell-derived CXCL13 is a crucial pathogenic factor in idiopathic multicentric Castleman disease
Source: Nat Commun. 2023 Oct 31;14:6959. doi: 10.1038/s41467-023-42718-0 (PMC10618253; doi:10.1038/s41467-023-42718-0)
Supplement: Supplementary file 1 — Supplementary Information [file 41467_2023_42718_MOESM1_ESM.pdf]

Supplementary Information

Peripheral helper-T-cell-derived CXCL13 is a crucial pathogenic factor in idiopathic multicentric Castleman disease

Takuya Harada[1,†], Yoshikane Kikushige[1,2,†],Toshihiro Miyamoto[3], Kazuko Uno[4], Hiroaki Niiro[1], Atsushi Kawakami[5], Tomohiro Koga[5], Koichi Akashi[1,2,*], and Kazuyuki Yoshizaki[6,7,*].

[1]Department of Medicine and Biosystemic Science, Kyushu University Graduate School of Medical Sciences, Fukuoka, Japan.

[2]Center for Cellular and Molecular Medicine, Kyushu University Hospital, Fukuoka, Japan.

[3]Department of Hematology, Faculty of Medicine, Institute of Medical Pharmaceutical and Health Sciences, Kanazawa University, Ishikawa, Japan

[4]Luis Pasteur Center for Medical Research, Kyoto, Japan.

[5]Department of Immunology and Rheumatology, Division of Advanced Preventive Medical Sciences, Nagasaki University Graduate School of Biomedical Sciences, Nagasaki, Japan.

[6]The Institute of Scientific and Industrial Research, SANKEN, Osaka University, Osaka, Japan.

[7]Medical corporation of Tokushukai, Osaka, Japan

[†] These authors contributed equally: Takuya Harada, Yoshikane Kikushige

*These authors jointly supervised this work: Koichi Akashi, Kazuyuki Yoshizaki

Supplementary Figures

Supplementary Fig.1: Histopathology images of LN of each patient with iMCD-NOS.
Supplementary Fig.2: Xenotransplantation of iMCD-NOS LN cells into NSG mice induced lethal inflammation *in vivo*.
Supplementary Fig.3: Polyclonal human B cells expanded in the spleen of iMCD-NOS NSG mice.
Supplementary Fig.4: T-B interaction was required for the development of iMCD-like systemic inflammation *in vivo*.
Supplementary Fig.5: Human Tph cells expanded in the iMCD-NOS NSG mice, whereas human FDC and Tfh cells did not.
Supplementary Fig.6: Improvement of serum albumin levels in the iMCD-NOS NSG mice treated with anti-hCXCL13 antibody and the presence of hCXCL13-expressing human Tph cells in iMCD-NOS NSG mice.

Supplementary Tables

Supplementary Table.1: Patient characteristics of iMCD and control.
Supplementary Table.2: Summary of xenogeneic transplantation assays of iMCD.

Supplementary Fig.1. Histopathology images of LN of each patient with iMCD-NOS

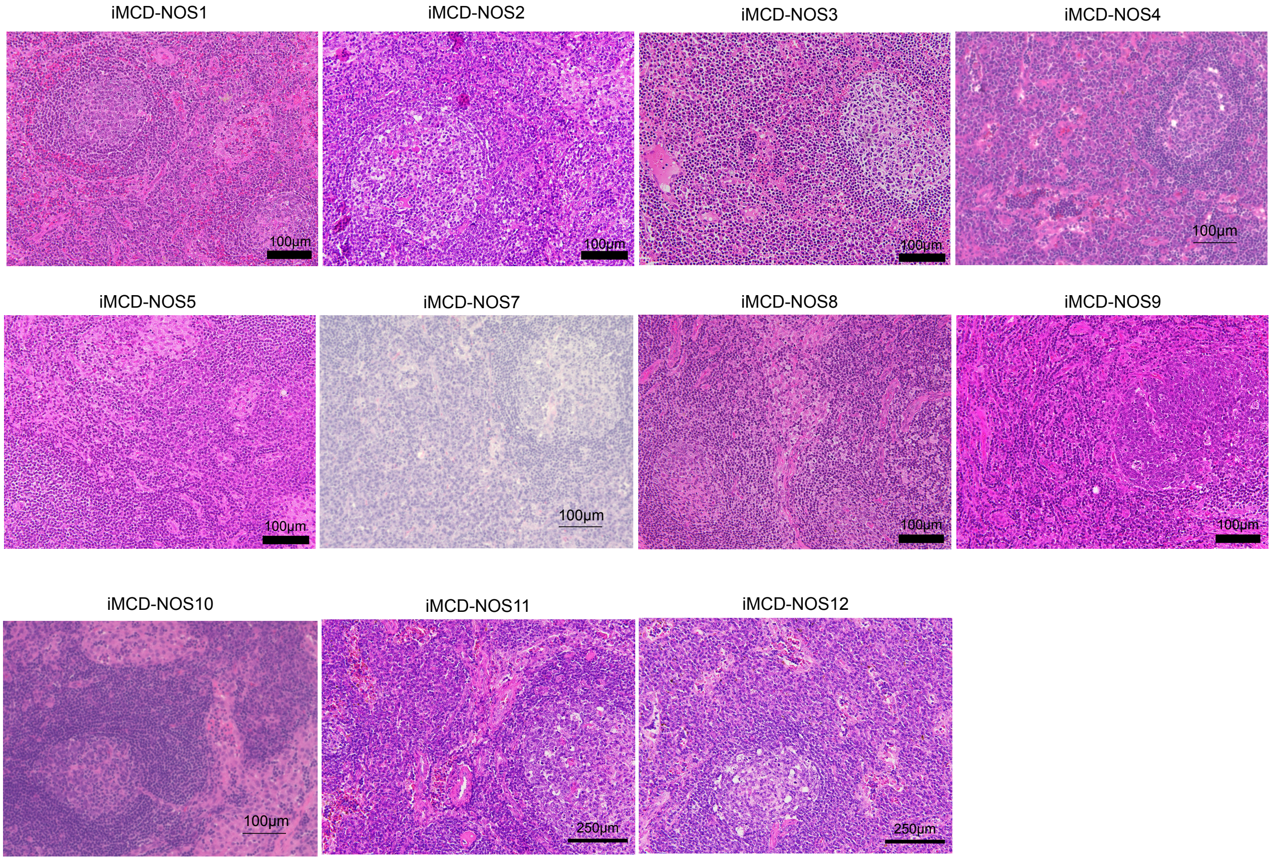

Histopathology images (HE staining) of iMCD-NOS LN samples used in the study are shown. Of note, we used only blood samples from the iMCD-NOS6 patient, so the histopathology image of iMCD-NOS6 is not shown. HE scale bar = 100 μm (P1, P2, P3, P4, P5, P6, P7, P8, P9 and P10) and 250 μm (P11 and P12).

Supplementary Fig.2. Xenotransplantation of iMCD-NOS LN cells into NSG mice induced lethal inflammation *in vivo*.

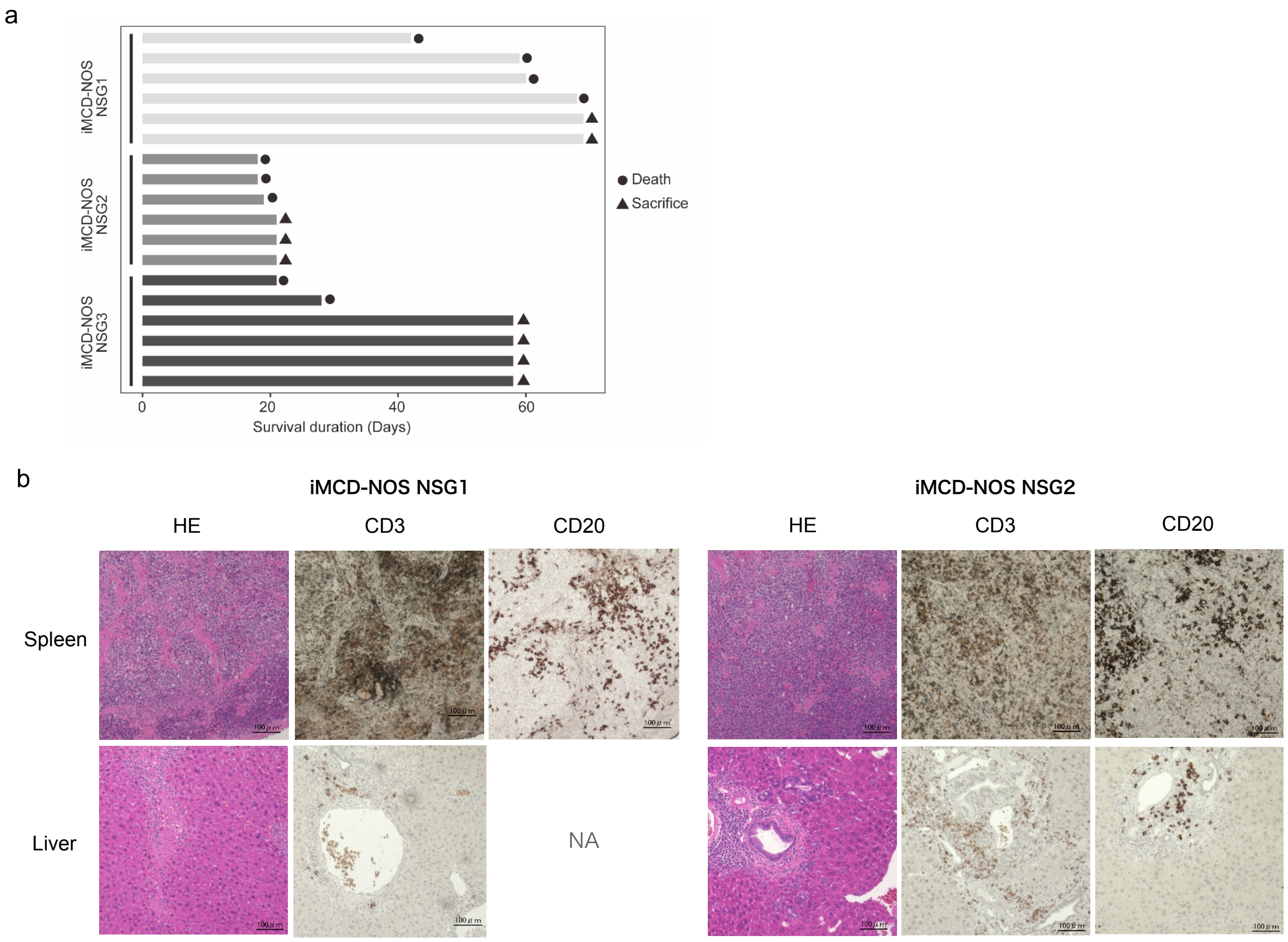

a. Swimmer plots of recipient mice transplanted with LN cells from patients with iMCD-NOS (P1, P2, and P3). Circles indicate cases of death. Triangles indicate cases with a clinical severity score (CSS) of 4 and sacrificed. **b.** Histological analysis of the spleen and liver of iMCD-NOS NSG1(P1) and iMCD-NOS NSG2(P2) mice. Immunostaining was repeated at least three times. Source data are provided as a Source Data. NA; not available

Supplementary Fig.3. Polyclonal human B cells expanded in the spleen of iMCD-NOS NSG mice.

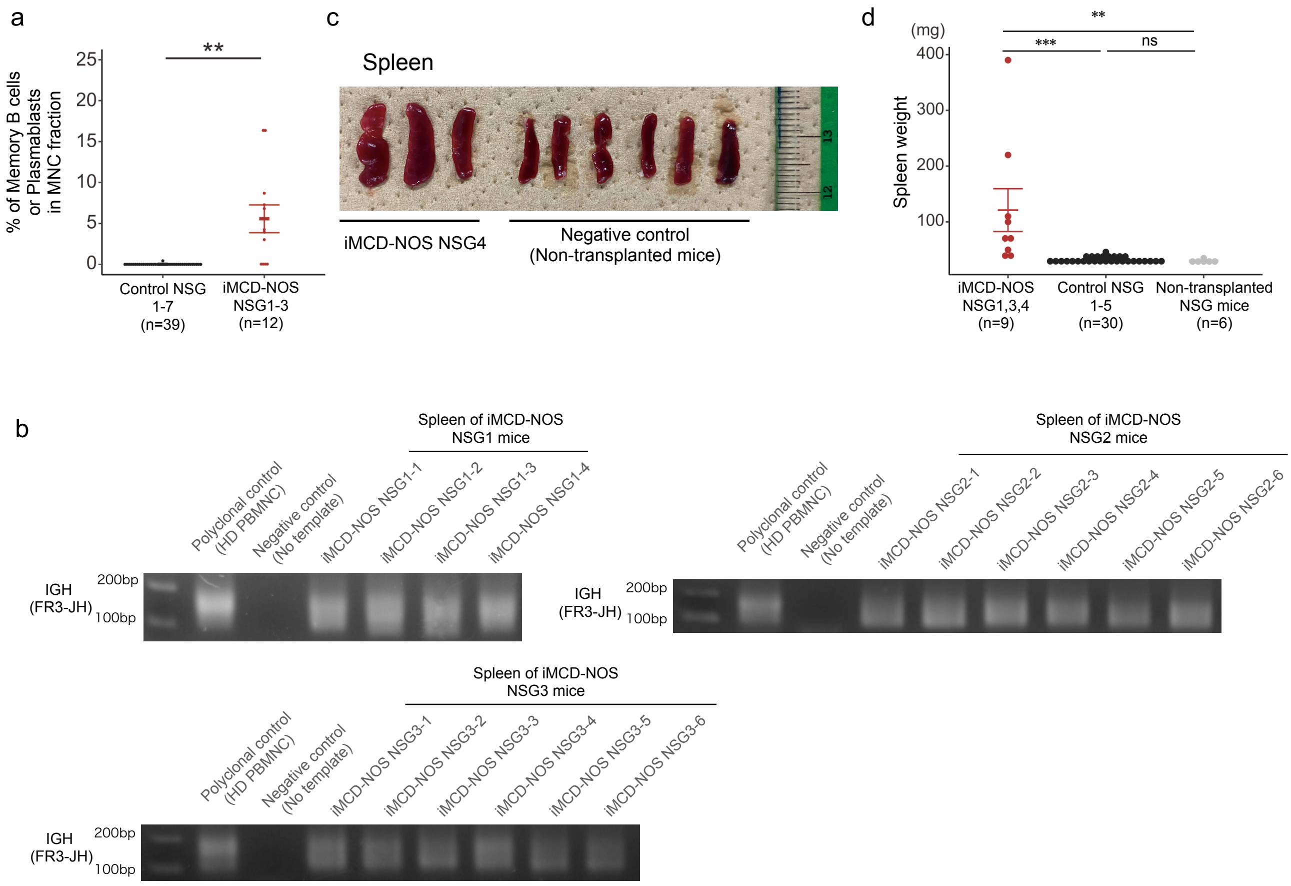

a. Percentage of Memory B cells or Plasmablasts in CD19^{+} fraction between Control NSG mice (n=39) and iMCD-NOS NSG mice (n=12). Comparisons between the two groups was analyzed with two-tailed unpaired *t*-test. (p = 0.007427) Data are represented as mean ± SE. **b.** Immunoglobulin heavy chain (IgH) rearrangement status of human B cells engrafted in the recipient mice (spleen). Polyclonal human B cell expansion was confirmed by PCR analysis. Peripheral blood obtained from the healthy donor was used as polyclonal control. **c.** Spleens of the NSG mice transplanted with iMCD-NOS4 (P4) were clearly enlarged compared to those of non-transplanted NSG mice. **d.** Spleen weight of iMCD-NOS NSG mice (1,3, and 4) were significantly increased compared to that of the control NSG mice (control 1-5: n=30 and non-transplanted NSG: n=6). Data are represented as means ± SD. *P* values were calculated using one-way ANOVA with Tukey posttest for multiple comparisons. **: $p < 0.01$ ***: $p < 0.001$, ns: not significant. (iMCD vs Control: $p = 0.0001013$, Non transplanted vs Control: $p = 0.9944532$, iMCD vs Non transplanted: $p = 0.0038686$). Source data are provided as a Source Data.

Supplementary Fig.4. T-B interaction was required for the development of iMCD-like systemic inflammation *in vivo*.

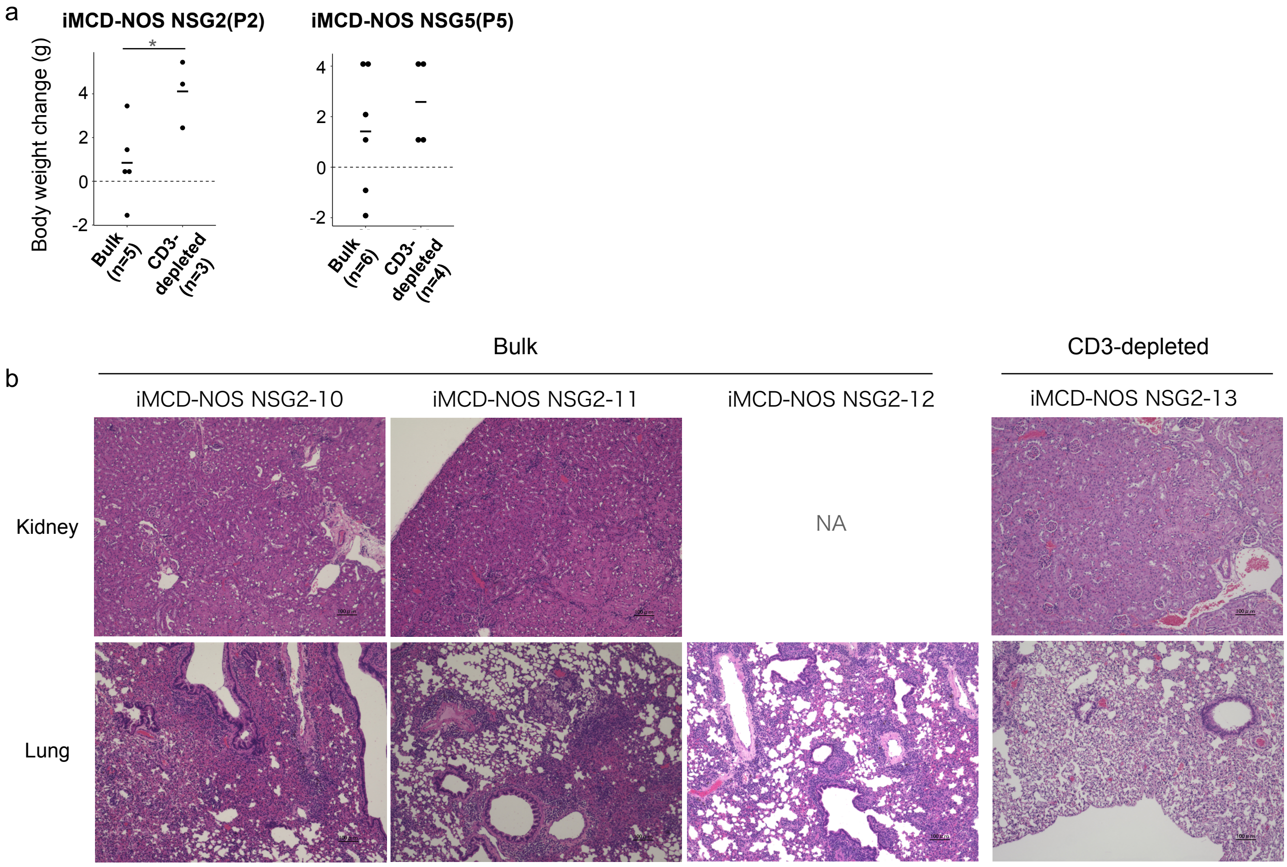

a. Body weight changes of iMCD-NOS NSG2 (bulk: n=5 and CD3-depleted: n=4) and iMCD-NOS NSG5 mice (bulk: n=6 and CD3-depleted: n=4) comparing from pre-transplantation at the time of 8 weeks after transplantation are presented. Comparisons between the two groups were analyzed with two-tailed unpaired *t*-test. (P2: p = 0.04134 and P5: p = 0.4094) **b.** Histological analysis of kidney (upper panels) and lung (lower panels) of the iMCD-NOS NSG2 mice transplanted with unmanipulated LN (left) and CD3-depleted LN (right) cells. NA; not available. *: $p< 0.05$. Source data are provided as a Source Data.

Supplementary Fig.5. Human Tph cells expanded in the iMCD-NOS NSG mice, whereas human FDC and Tfh cells did not.

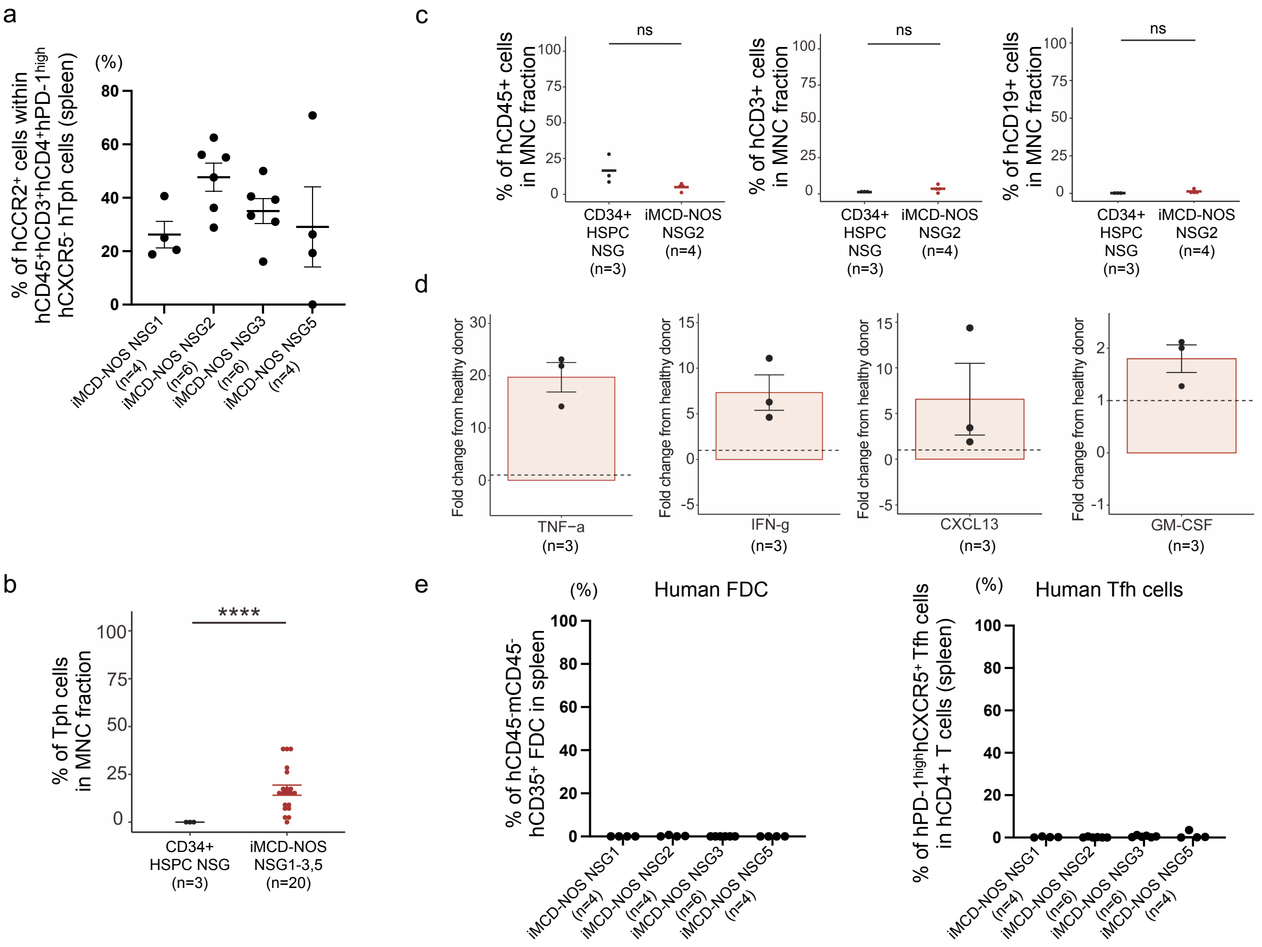

a. The frequencies of hCCR2$^+$ Tph cells in the spleen of the iMCD-NOS NSG mice (NSG1: n=4, NSG2: n=6, NSG3: n=6 and NSG5: n=4) are shown. Data are shown as mean ± SE. **b.** The percentage of Tph cells in mononuclear cells fraction between humanized NSG mice (CD34$^+$ HSPC transplanted NSG mice, n=3) and iMCD-NOS NSG mice (n=20). Comparison between the two groups was analyzed with two-tailed unpaired *t*-test. (p = 4.406 x 10^{-6}) Data are represented as mean ± SE. **c.** The frequencies of human CD45$^+$ cells, CD3$^+$ cells and CD19$^+$ cells between humanized NSG mice (CD34$^+$ HSPC transplanted NSG mice, n=3) and iMCD-NOS NSG (n=4). Comparisons between the two groups were analyzed with two-tailed unpaired *t*-test. (CD45: p = 0.1825, CD3: p = 0.1714, CD19: p = 0.1748) **d.** The iMCD-NOS patient (P2) exhibited the elevated ratio of TNF-a, IFN-g, CXCL13 and GM-CSF levels in the serum as compared to three independent healthy donors (n=3). Data are shown as mean ± SE; dotted line indicates 1. The values for the patient sample were ratioed with the values for three independent healthy samples, and the mean ± SE of the calculated values are shown as error bars. The value of mean - SE greater than 1.0 indicates that the patient sample values are significantly higher than the population of healthy samples. **e.** The frequencies of human FDC (left, iMCD-NOS NSG1: n=4, NSG2: n=4, NSG3: n=6, and NSG5: n=4) and Tfh cells (right, iMCD-NOS NSG1: n=4,

NSG2: n=6, NSG3: n=6, and NSG5: n=4) in the spleen of iMCD-NOS NSG mice are shown. ****: $p < 0.001$, n.s: not significant. Source data are provided as a Source Data.

Supplementary Fig.6. Improvement of serum albumin levels in the iMCD-NOS NSG mice treated with anti-hCXCL13 antibody and the presence of hCXCL13-expressing human Tph cells in iMCD-NOS NSG mice

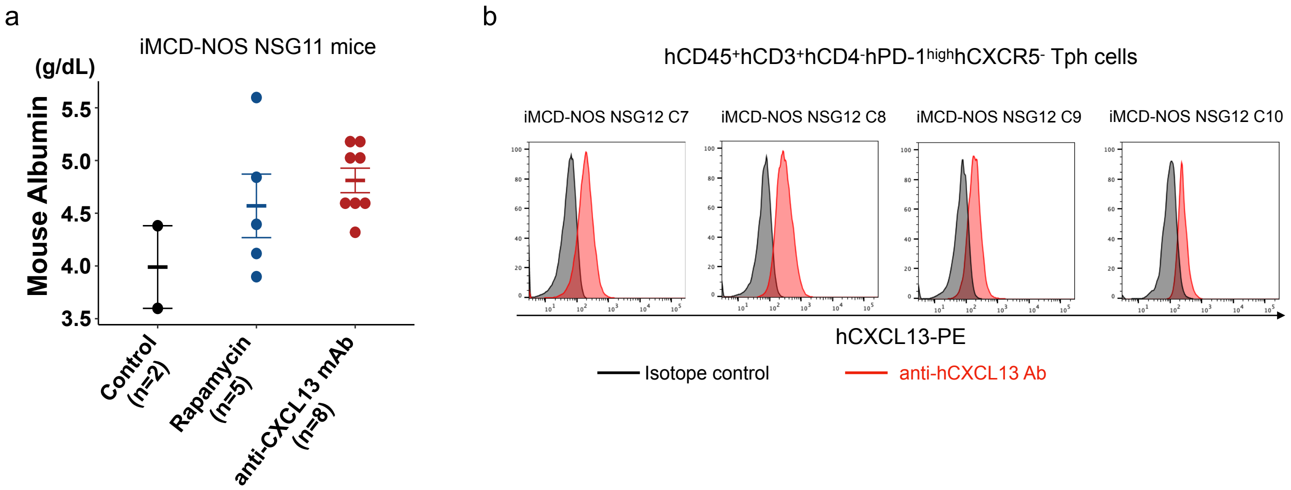

a. Mouse serum albumin levels at 8 weeks after transplantation are evaluated. Data represented as means ± SE. **b**. Intracellular staining results of hCXCL13 in human Tph cells in the four independent control iMCD-NOS NSG12 mice are shown. Source data are provided as a Source Data.
