# Supplementary material for: Peripheral helper-T-cell-derived CXCL13 is a crucial pathogenic factor in idiopathic multicentric Castleman disease
Source: Nat Commun. 2023 Oct 31;14:6959. doi: 10.1038/s41467-023-42718-0 (PMC10618253; doi:10.1038/s41467-023-42718-0)
Supplement: Supplementary file 5 — Reporting Summary [file 41467_2023_42718_MOESM5_ESM.pdf]

nature portfolio

Corresponding author(s): Kazuyuki Yoshizaki, MD, PhD and Koichi Akashi, MD, PhD.

Last updated by author(s): Oct 5, 2023

Reporting Summary

Nature Portfolio wishes to improve the reproducibility of the work that we publish. This form provides structure for consistency and transparency in reporting. For further information on Nature Portfolio policies, see our Editorial Policies and the Editorial Policy Checklist.

Statistics

For all statistical analyses, confirm that the following items are present in the figure legend, table legend, main text, or Methods section.

n/a	Confirmed	
☐	☒	The exact sample size (*n*) for each experimental group/condition, given as a discrete number and unit of measurement
☐	☒	A statement on whether measurements were taken from distinct samples or whether the same sample was measured repeatedly
☐	☒	The statistical test(s) used AND whether they are one- or two-sided<br>*Only common tests should be described solely by name; describe more complex techniques in the Methods section.*
☐	☒	A description of all covariates tested
☐	☒	A description of any assumptions or corrections, such as tests of normality and adjustment for multiple comparisons
☐	☒	A full description of the statistical parameters including central tendency (e.g. means) or other basic estimates (e.g. regression coefficient) AND variation (e.g. standard deviation) or associated estimates of uncertainty (e.g. confidence intervals)
☐	☒	For null hypothesis testing, the test statistic (e.g. *F*, *t*, *r*) with confidence intervals, effect sizes, degrees of freedom and *P* value noted<br>*Give P values as exact values whenever suitable.*
☒	☐	For Bayesian analysis, information on the choice of priors and Markov chain Monte Carlo settings
☒	☐	For hierarchical and complex designs, identification of the appropriate level for tests and full reporting of outcomes
☒	☐	Estimates of effect sizes (e.g. Cohen's *d*, Pearson's *r*), indicating how they were calculated

Our web collection on statistics for biologists contains articles on many of the points above.

Software and code

Policy information about availability of computer code

Data collection	Quantification of flowcytometry data of human lymph node cells, human peripheral mononuclear cells and mouse tissues cells were performed by BD FACS Aria II (BD Biosciences), BD FACS Aria IIIu (BD Biosciences), or Attune NxT Flow Cytometer (Thermo Fisher Scientific). These flowcytometry data were analyzed with the FlowJo software (version 10.7.1)<br><br>The images of HE stain or immunohistochemistory were captured using BZ-X710 (KEYENCE, software BZ-H3A)<br><br>The data of mass spectrometry (MS) were analyzed using Hyperion Imaging System (Fluidgm). The image of MS were captured using MCD viewer (ver 1.0.560.2)<br><br>CXCL13, immunoglobulins and mouse albumin were quantified by enzyme-linked immunoassay (ELISA) in a subset of samples of human blood serum and mouse blood serum. (CXCL13:ab269370, human IgG:ab195215, IgA:ab196263, IgE:ab195216, mouse albumin:ab108792)<br>Absorbance measurements were read using a microplate readere (MULTISKAN FC, Thermo Fisher Scientific)<br>Other cytokines and chemokines were catured and analyzed with Bio-Plex Suspension Array System (Bio-Rad Laboratories Inc.)
Data analysis	Statistical analyses and figure generation were performed using the R v4.0.3 or Graph pad Prism v9.5.0. Analysis was performed through previously published and publicly available R packages:gplots v3.1.1, dplyr v1.0.2, ggplot2 v3.3.3, readxl v1.3.1, survival v3.2-7, survminer v0.4.8.

For manuscripts utilizing custom algorithms or software that are central to the research but not yet described in published literature, software must be made available to editors and reviewers. We strongly encourage code deposition in a community repository (e.g. GitHub). See the Nature Portfolio guidelines for submitting code & software for further information.

Data

Policy information about availability of data

All manuscripts must include a data availability statement. This statement should provide the following information, where applicable:

- Accession codes, unique identifiers, or web links for publicly available datasets
- A description of any restrictions on data availability
- For clinical datasets or third party data, please ensure that the statement adheres to our policy

Original images used in the current study are uploaded at Mendeley Data, V1, doi: 10.17632/y6gtwwdsfx.1
The source data generated in this study for figures are provide in the Source Data file.

Research involving human participants, their data, or biological material

Policy information about studies with human participants or human data. See also policy information about sex, gender (identity/presentation), and sexual orientation and race, ethnicity and racism.

Reporting on sex and gender	Information on sex was collected from medical records. Information on gender was not collected. No sex-based analysis were performed.
Reporting on race, ethnicity, or other socially relevant groupings	Race/ethnicity of each participants was not used for analysis in this study.
Population characteristics	Samples were collected from a population of patients with iMCD-NOS.<br>All patients were diagnosed pathologically or clinically with iMCD-NOS by a specialist with diagnostic experience. Detailed patient characteristics are described in Supplementary Table1.<br><br>Lymph nodes of other control group cases (N = 9) were resected for the purpose of diagnosing enlarged lymph nodes. Consent was obtained from all participant of control group at Kyushu University Hospital or Tokushukai Group Hospital.
Recruitment	iMCD patients were selected based on the clinical diagnosis by the physician who experienced iMCD diagnosis from among patients looked in an outpatient clinic. Therefore, the cases of iMCD selected in this study are a relatively homogeneous group of cases.<br><br>However, It is possible that cases with laboratory abnormalities which are clinically suggestive of iMCD were selected, which might have a selection bias.
Ethics oversight	Kyushu university Institutional Review Board (2020-576), Tokushukai Group Institutional Review Board (TGE01887-004).

Note that full information on the approval of the study protocol must also be provided in the manuscript.

Field-specific reporting

Please select the one below that is the best fit for your research. If you are not sure, read the appropriate sections before making your selection.

☒ Life sciences ☐ Behavioural & social sciences ☐ Ecological, evolutionary & environmental sciences

For a reference copy of the document with all sections, see nature.com/documents/nr-reporting-summary-flat.pdf

Life sciences study design

All studies must disclose on these points even when the disclosure is negative.

Sample size	Sample size calculations for the creation of PDX mice were not calculated since idiopathic multicentric Castleman disease (iMCD-NOS) is a rare disease.<br>For in vivo and human sample experiments, the sample size was determined to be sufficient to obtain the statistical difference between groups. Due to statical analysis, there were at least three samples in a experiment.<br><br>Lymph node biopsy specimens for the purpose of diagnosing iMCD-NOS were used, and stored specimens of these were used to create PDX mice. One case was biopsied at the time of relapse of iMCD-NOS after tocilizumab treatment. This study included 12 cases of iMCD-NOS.<br><br>Reactive lymph node (N = 3) or lymphoma specimens (N = 4) were used as a control group to generate PDX mice. Since those cells were not viable in NSG mice, cord blood (N = 3) was used to create PDX mice for the purpose of reproducing human steady-state cytokines/chemokines. In addition, other reactive lymph nodes (N = 2) were used for flowcytometry analysis of Tph cells.
Data exclusions	Tissues from mice euthanized or died after transplantation by the protocol were extracted and analyzed for human hematopoietic cells by flowcytometory.<br>Some cases exist where viable tissue could not be extracted because of the time required for postmortem cell extraction. (iMCD-NOS1, bone

marrow: N = 2, iMCD-NOS1, spleen: N=2, iMCD-NOS2, bone marrow: N = 3, iMCD-NOS2, spleen: N = 1, iMCD-NOS3, bone marrow: N = 2, iMCD-NOS4, bone marrow: N = 6, iMCD-NOS4, spleen: N = 6, iMCD-NOS5, bone marrow: N = 3, iMCD-NOS5, spleen: N = 2, Control 4, spleen: N = 1)

Due to the lack of stored specimens in some cases, we were unable to analyze the percentage of MemoryB cells or Plasmablast(Fig2b), Tph cells (Fig4b), FDC cells (FigS5c) and TFH (FigS5c). (MemoryB or Plasmablast: iMCD-NOS1 N = 3, iMCD-NOS2 N = 1, iMCD-NOS3 N = 2, Control4 N = 2, Control5 N = 1, Tph: iMCD-NOS1 N = 2, iMCD-NOS5 N = 4, FDC: iMCD-NOS1 N = 2, iMCD-NOS2 N=2, iMCD-NOS5 N = 4, TFH: iMCD-NOS1 N = 2, iMCD-NOS5 N = 4)

In addition, some cases were observed in which cardiac blood sampling for mouse serum extraction was difficult. (Fig3c: iMCD-NOS2 N = 1, Fig3d: iMCD-NOS5 N = 3)

Replication

All figures presented with error bars were derived from at least three independent experiments. The exact numbers of the samples and experiments were shown in Figures and Figure legends.

Since the volumes of samples including serum and reconstituted human cells in NSG mice were limited, the reproducibility was confirmed by comparing samples from three independent cases in the grouped mice.

Randomization

In vivo treatment experiments, the recipient NSG mice were randomly allocated into two or three groups with equivalent body weight.

Blinding

All in vivo experiments were performed in blinding except for the treatment experiments to PDX mice.

With respect to the treatment analysis, we could not be blinded in terms of grouping the treatment groups.

Serum analysis and flow cytometry analysis were performed blindly.

Reporting for specific materials, systems and methods

We require information from authors about some types of materials, experimental systems and methods used in many studies. Here, indicate whether each material, system or method listed is relevant to your study. If you are not sure if a list item applies to your research, read the appropriate section before selecting a response.

Materials & experimental systems

n/a	Involved in the study
☐	☒ Antibodies
☒	☐ Eukaryotic cell lines
☒	☐ Palaeontology and archaeology
☐	☒ Animals and other organisms
☒	☐ Clinical data
☒	☐ Dual use research of concern
☒	☐ Plants

Methods

n/a	Involved in the study
☒	☐ ChIP-seq
☐	☒ Flow cytometry
☒	☐ MRI-based neuroimaging

Antibodies

Antibodies used

The following anti-mouse primary antibodies were used: anti-CD45 (30-F11, BioLegend, 1:50) and Ter119 (TER-119, BioLegend, 1:50). The following anti-human antibodies were used: anti-CD3 (UCHT1, BioLegend, primary, 1:50), anti-CD4 (RPA-T4, BioLegend, primary, 1:50), anti-CD8a (RPA-T8, BioLegend, primary, 1:50), anti-CD10 (HI10a, Biolegend, primary, 1:50), anti-CD19 (HIB19, BioLegend, primary, 1:50), anti-CD20 (2H7, BioLegend, primary, 1:50), anti-CD27 (M-T271, BioLegend, primary, 1:50), anti-CD34 (8G12, BD Biosciences, primary, 1:50), anti-CD35 (E11, BioLegend, primary,1:50), anti-CD38 (HIT2, BioLegend, primary, 1:50), anti-CD45 (HI30, BioLegend, 1:50), anti-CD185(CXCR5) (J252D4, BioLegend, primary, 1:20), anti-CD192 (CCR2) (K036C2, BioLegend, primary, 1:20), anti-CXCL13(IC801P and IC8012R from R&D Systems for primary antibody, 1:10, and A15151D from BioLegend for primary antibody, 1:10) and anti-CD279 (PD-1) (EH12.2H7, BioLegend, primary, 1:20).
The following antibodies for imaging mass cytometry (IMC) were used: anti-human CD4 (EPR6855, cat# 3156033D, Fluidigm, 156Gd, 1:100), anti-human CD8a (C8/144B, cat# 3162034D, Fluidigm, 162Dy, 1:50) and anti-human CD20 (H1, cat# 3161029D, Fluidigm, 161Dy, 1:200).

Validation

Appropriate FMO staining and isotype controls were used to determine the negative line for FCM.
Technical data-sheet for anti-CD45: https://www.biolegend.com/ja-jp/products/purified-anti-mouse-cd45-antibody-102
Technical data-sheet for Ter119: https://www.biolegend.com/ja-jp/products/percp-cyanine5-5-anti-mouse-ter-119-erythroid-cells-antibody-4292?GroupID=ImportedGROUP1
Technical data-sheet for anti-CD3: https://www.biolegend.com/ja-jp/products/purified-anti-human-cd3-antibody-867?GroupID=BLG5900
Technical data-sheet for anti-CD4: https://www.biolegend.com/ja-jp/products/purified-anti-human-cd4-antibody-830?GroupID=BLG7755
Technical data-sheet for anti-CD8a: https://www.biolegend.com/ja-jp/products/purified-anti-human-cd8a-antibody-839?GroupID=BLG5903
Technical data-sheet for anti-CD10: https://www.biolegend.com/en-us/products/purified-anti-human-cd10-antibody-2214?GroupID=BLG5905
Technical data-sheet for anti-CD19: https://www.biolegend.com/ja-jp/products/purified-anti-human-cd19-antibody-721

Technical data-sheet for anti-CD20: https://www.biolegend.com/ja-jp/products/purified-anti-human-cd20-antibody-561?GroupID=BLG7904
Technical data-sheet for anti-CD27: https://www.biolegend.com/ja-jp/products/purified-anti-human-cd27-antibody-8341?GroupID=BLG10174
Technical data-sheet for anti-CD34: https://www.bdbiosciences.com/ja-jp/products/reagents/flow-cytometry-reagents/clinical-discovery-research/single-color-antibodies-ruo-gmp/pe-mouse-anti-human-cd34.348057
Technical data-sheet for anti-CD35: https://www.biolegend.com/ja-jp/products/purified-anti-human-cd35-antibody-4676?GroupID=BLG10176
Technical data-sheet for anti-CD38: https://www.biolegend.com/ja-jp/products/purified-anti-human-cd38-antibody-748?GroupID=BLG10099
Technical data-sheet for aniti-CD45 :https://www.biolegend.com/ja-jp/cell-health/purified-anti-human-cd45-antibody-710?GroupID=BLG5926
Technical data-sheet for anti-CD185(CXCR5): https://www.biolegend.com/ja-jp/products/purified-anti-human-cd185-cxcr5-antibody-8357
Technical data-sheet for anti-CD192(CCR2): https://www.biolegend.com/ja-jp/products/purified-anti-human-cd192-ccr2-antibody-8383?GroupID=BLG11385
Technical data-sheet for PE-conjugated anti-human CXCL13 (R&D Systems): https://www.rndsystems.com/products/human-cxcl13-blc-bca-1-pe-conjugated-antibody-53610_ic801p
Technical data-sheet for Alexa Fluor 647-conjugated anti-human CXCL13 (R&D Systems): https://www.rndsystems.com/products/human-cxcl13-blc-bca-1-alexa-fluor-647-conjugated-antibody-53602_ic8012r
Technical data-sheet for anti-CXCL13 (Biolegend): https://www.biolegend.com/ja-jp/products/ultra-leaf-purified-anti-human-cxcl13-blc-antibody-19082
Technical data-sheet for anti-CD279 (PD-1): https://www.biolegend.com/ja-jp/products/purified-anti-human-cd279-pd-1-antibody-4410
Technical data-sheet for anti-CD4 for IMC: https://fluidigm.my.salesforce.com/sfc/p/#700000009DAw/a/4u0000019q09/z61I1lPUa.yt50BzirMZu6pIxE8QwiOjQmvMkdCf6pg
Technical data-sheet for anti-CD8a for IMC: https://fluidigm.my.salesforce.com/sfc/p/#700000009DAw/a/4u0000019iPY/ZRRsUI7mndudRs75P5yHNEgOiXemHRb0KlSPirqxpQ4
Technical data-sheet for anti-CD20 for IMC: https://fluidigm.my.salesforce.com/sfc/p/#700000009DAw/a/4u0000019iPn/IeuPSkutkaZ_753BAzJyvphzfzhkDEnWSNGRZs.9lIg

Animals and other research organisms

Policy information about studies involving animals; ARRIVE guidelines recommended for reporting animal research, and Sex and Gender in Research

Laboratory animals	Female NSG (NOD.Cg-PrkdcscidIl2rgtm1Wjl/Sz) mice at 4–6 weeks old were purchased from Charles River Laboratories Japan, and were bred and housed in a specific pathogen-free facility in micro-isolator cages at Kyushu University. The following conditions were controlled: daily light period from 07:00 to 19:00, temperature at 23±1 degree celsius and humidity at 50% with free access to water and food.
Wild animals	This study did not involve the wild animals.
Reporting on sex	Due to efficient engraftment rate of human hematological cells in female NSG mice rather than male NSG mice, only female NSG mice were used in this study.
Field-collected samples	This study did not involve the field-collected samples.
Ethics oversight	Animal experiments were performed in accordance with institutional guidelines approved by the Animal Care Committee of Kyushu University (A23-291-0).

Note that full information on the approval of the study protocol must also be provided in the manuscript.

Flow Cytometry

Plots

Confirm that:

☒ The axis labels state the marker and fluorochrome used (e.g. CD4-FITC).

☒ The axis scales are clearly visible. Include numbers along axes only for bottom left plot of group (a 'group' is an analysis of identical markers).

☒ All plots are contour plots with outliers or pseudocolor plots.

☒ A numerical value for number of cells or percentage (with statistics) is provided.

Methodology

Sample preparation	The lymph nodes were minced into 1–3 mm3 fragments in RPMI 1640 supplemented with 10% fetal bovine serum (FBS). The floating cells were collected from the medium.<br>In PDX assays, spleen and liver were putted in the cell strainer (100um) and grinded with a syringe plunger using 10mL of PBS to pass through the strainer.<br>Bone marrow cells were isolated from femurs using PBS and 25-gauge needle to wash.

All collected samples from mice were centrifuged at 400g for 5min at 4 degree celsius. After centrifugation, supernatants were completely discarded and cell pellets were hemolyzed using BD Pharm LyseTM lysisng Buffer. (Technical data-sheet:https://www.bdbiosciences.com/ja-jp/products/reagents/cell-preparation-separation-reagents/blood-lysis/staining-and-cell-preparation/lysing-buffer.555899)

All samples after hemolysis were centrifuged at 400g for 5min at 4 degree celsius. After centrifugation, supernatants were completely discarded and cell pellets were resuspended in stain buffer (HBSS with 2mM EDTA and 1% FBS). All cell suspensions were incubated for 15min with Fc receptor blocking reagent.

In cell surface staining, after washing and resuspension using stain buffer, all cell suspensions were incubated for 30min with fluorescent dye-labeled antibodies on ice, kept away from light. After incubation, cells were washed once using 1ml stain buffer with a centrifugation (400g, 5min, 4 degree celsius) and resuspended in stain buffer containing Propidium Iodide.

In intracellular staining, cell surface staining was performed before intracellular cytokine staining for 20min. After washing two times, fixation/permeabilization buffer (BD Biosciences) was added to fix the cells for 20min. Antibodies to detect CXCL13 (Biolegend) were added to cell suspension and intracellular staining was performed for 15min. After washing two times, cells were analyzed by BD FACS Aria II (BD Biosciences), BD FACS Aria IIIu (BD Biosciences), or Attune NxT Flow Cytometer (Thermo Fisher Scientific).

Instrument

BD FACS Aria II (BD Biosciences), BD FACS Aria IIIu (BD Biosciences), or Attune NxT Flow Cytometer (Thermo Fisher Scientific)

Software

FlowJo software (version 10.7.1)

Cell population abundance

At least 7000 events were acquired for cells in each assay.

Gating strategy

Cells were initially gated over the 2D density of events on forward and side scatter (FSC-H/SSC-H) to exclude cell debris. Subsequently, live cells without mouse erythrocytes were gated on absence of Propidium Iodide staining and Ter119 staining. Doublets cells were discriminated using the 2D density of events on FSC-H/FSC-W and SSC-H/SSC-W. Downstream gating is described as analysis of mono nuclear cells (MNC) in Supplementary Table2.

☒ Tick this box to confirm that a figure exemplifying the gating strategy is provided in the Supplementary Information.
